# Cultivation of Medicinal Plants in Ohio

**Published:** 1834

**Authors:** 


### III.—Cultivation of Medicinal Plants in Ohio.

The society of *Shakers* at Union Village, Warren County, Ohio, cultivate and prepare a variety of medicinal plants—native and exotick. This is a branch of horticulture in which the profession is interested, and the industrious and orderly community who have undertaken it, deserve encouragement. Orders to be directed to A. C. Houston.

